# Vaccines work… and do not cause autism

**DOI:** 10.1371/journal.pbio.3003143

**Published:** 2025-04-18

**Authors:** Nonia Pariente, on behalf of the PLOS Biology staff editors

**Affiliations:** Public Library of Science, San Francisco, California, United States of America and Cambridge, United Kingdom

## Abstract

Vaccines have saved millions of lives, yet their importance and safety is repeatedly in question. W e cannot let disinformation campaigns get in the way of global public health – it is not time to defund, but rather invest in these life-saving tools.

Louis Pasteur said, a tad optimistically, that “it is within the power of man to eradicate infection from the earth” [1]. That power, harnessed through vaccination, has so far eradicated two viruses: smallpox and rinderpest (which is closely related to measles). We were quite close to eradicating several others, but COVID-19-related disruptions, vaccine hesitancy and, more recently, vaccine defunding, are endangering our progress.

It beggars belief that some of the social discourse has become so ideologically tainted and hostile, as vaccines are on the whole very safe and extensively tested. It is tragic that during the COVID-19 pandemic, excess mortality in the US was significantly higher among Republicans than Democrats after vaccines were available, but not before [2], or that two people have recently died of measles in the midst of a growing US outbreak, where cheap, safe and effective vaccines are readily available. Recent moves to strip funding from vaccine-related research and possibly mRNA technology (which is promising not only against infectious disease, but also against cancer [3]) seem shortsighted and self-harming. As does the use of precious resources to revisit debunked claims of links to autism, which is known to fuel societal concern [4]. Globalizing antivaccine activism could become our greatest hurdle.

Let me be clear, there can be no doubt that vaccines are one of the greatest scientific success stories of our time. Global vaccination efforts have saved at least 154 million lives in the last 50 years [5]—six lives every minute of every year. Immunization against measles accounts for 60% of the lives saved, and vaccination against 14 other diseases has reduced infant deaths by 40% globally, and over 50% in Africa [5]. And this is without taking into account the many lives saved by the COVID-19 vaccines or the HPV vaccine, which could eliminate cervical cancer. New vaccines, such as those for malaria, respiratory syncytial virus, meningitis, cholera and Ebola virus, will contribute to saving more lives.

And yet it feels like the importance of funding vaccine research and supporting global vaccination efforts needs to be justified over and over again. As increasing outbreaks and new zoonoses continue to remind us, nobody is safe from disease until we are all safe, and vaccines are one of the best tools we have to control pathogens. Our technical ability to develop vaccines has outpaced our social, political, and financial systems for ensuring their use.

This month, in which the World Health Organization celebrates “World Immunization Week”, we feature two Perspective articles on the topic of vaccines [6, 7]. In the first, Sarah Gilbert—whose work contributed to the development of the Oxford-AstraZeneca COVID-19 vaccine—discusses priorities for effective development and deployment of vaccines against outbreak infections, as well as the acceleration that vaccine platforms have brought to new vaccine development [6]. In the second, Andrea Marzi—who develops vaccines against filoviruses (such as Ebola and Marburg virus)—argues for the need to develop vaccines that can afford protection across different types of Ebola virus, and even across all filoviruses [7].

Vaccination technology and logistics have come a long way since the Balmis Expedition set off from my hometown of A Coruña in 1803 to take the Jenner smallpox vaccine to the Americas and beyond, by passing it from arm to arm of 22 local orphans during the Atlantic crossing [8]. It is estimated that this expedition immunized 300,000 people in 3 years, arguably the first large-scale mass vaccination of its kind [8]. By comparison, in the 3.5 years after December 2020, over 13.5 billion doses of COVID-19 vaccines were administered across the World. And yet, considerable work remains to be done if we are to be prepared for the next outbreak, as Sarah Gilbert explains [6].

Vaccine platforms, so useful in the COVID-19 pandemic, provide a known chassis in which to quickly introduce new antigens, but more research is needed to expand these approaches to other pathogens. For example, although new platforms are being developed to manufacture flu vaccines, the great majority are still produced in chicken eggs—a laborious, costly and slow technology first licensed in 1945 [9]. We all seem accustomed to flu, but seasonal influenza virus infection still kills up to 650,000 people a year globally, and there have been at least six pandemic outbreaks, with the 1918 flu killing almost 20 million people. The current H5N1 pandemic in birds, cattle, and 50 mammalian species causes particular concern, as it could evolve to transmit from human to human [10]. Given that influenza virus is highly variable and requires frequent vaccine updates, it would be important to be able to pivot quickly and produce them rapidly at scale.

The degree to which vaccines can provide cross-protection, that is, prevent disease caused by related viruses, is also important. Andrea Marzi discusses the need for vaccines that can protect from a range of filoviruses [7]. These viruses cause increasingly frequent outbreaks in Africa, leading to localized public health emergencies and sometimes large transnational outbreaks of hemorraghic fever. Having a vaccine at the ready, effective against a broad swathe of viruses (or several vaccines effective against a smaller subset of viruses) would be instrumental for quick responses that prevent local outbreaks from spiralling out of control. Durable cross-protection, or even universal protection, would also be a Holy Grail in flu vaccine research, as it would avoid the need for constant reformulation to catch up with virus evolution.

Importantly, as Gilbert notes, if we don’t manufacture, cannot deploy or afford a vaccine, then we can’t vaccinate [6]. Vaccine stability in the absence of a cold chain matters. Vaccine supply matters. Cost per dose matters. Vaccine effectiveness is not the same as vaccine efficacy, and is arguably much more important. The best vaccine isn’t useful if it is not distributed, or not socially accepted. Vaccine equity and hesitancy remain great challenges that we ignore at our peril [11].

There is no single approach that will work for every disease, and we still don’t have effective vaccines for major killers, such as HIV-1, not to mention major bacterial and fungal diseases. With H5N1 influenza spreading, measles cases on the rise, COVID-19 still circulating, an Ebola outbreak in Uganda and Mpox having been detected in 127 countries, it clearly is not the time to defund, but rather to invest in vaccines.

We should not allow misinformation, disinformation, ignorance and defunding to hinder vaccine development, deployment and use. We need all hands on deck to defend the importance of vaccine research and the safety of vaccines. Go forth and spread the word!
